# Prospects for comparison of matter and antimatter gravitation with ALPHA-g

**DOI:** 10.1098/rsta.2017.0265

**Published:** 2018-02-19

**Authors:** W. A. Bertsche

**Affiliations:** 1 University of Manchester, School of Physics and Astronomy, Oxford Road, Manchester M13 9PL, UK; 2 The Cockcroft Institute, Keckwick Lane, Daresbury, Warrington WA4 4AD, UK

**Keywords:** gravity, antimatter, CPT, Lorentz invariance, antigravity, antihydrogen

## Abstract

The ALPHA experiment has recently entered an expansion phase of its experimental programme, driven in part by the expected benefits of conducting experiments in the framework of the new AD + ELENA antiproton facility at CERN. With antihydrogen trapping now a routine operation in the ALPHA experiment, the collaboration is leading progress towards precision atomic measurements on trapped antihydrogen atoms, with the first excitation of the 1S–2S transition and the first measurement of the antihydrogen hyperfine spectrum (Ahmadi *et al.* 2017 *Nature* **541**, 506–510 (doi:10.1038/nature21040); *Nature* **548**, 66–69 (doi:10.1038/nature23446)). We are building on these successes to extend our physics programme to include a measurement of antimatter gravitation. We plan to expand a proof-of-principle method (Amole *et al.* 2013 *Nat. Commun.* **4**, 1785 (doi:10.1038/ncomms2787)), first demonstrated in the original ALPHA apparatus, and perform a precise measurement of antimatter gravitational acceleration with the aim of achieving a test of the weak equivalence principle at the 1% level. The design of this apparatus has drawn from a growing body of experience on the simulation and verification of antihydrogen orbits confined within magnetic-minimum atom traps. The new experiment, ALPHA-g, will be an additional atom-trapping apparatus located at the ALPHA experiment with the intention of measuring antihydrogen gravitation.

This article is part of the Theo Murphy meeting issue ‘Antiproton physics in the ELENA era’.

## 1. Introduction

Antimatter and gravity are central players in two of the great outstanding debates in physics today. In the first, researchers are trying to understand the observed excess of matter over antimatter in the Universe today. In the second, the fundamental nature of gravity and its connection to quantum mechanics, or a grand unified theory in general, remain elusive. Precision studies of antihydrogen atoms provide an opportunity to probe the first question generally, and may provide specific insight for the second.

As a platform for comparing matter and antimatter properties, antihydrogen is a useful system to study. Few physical systems have been studied both experimentally and theoretically as extensively or at such high precision as the hydrogen atom. Its simplicity makes this effort both possible as well as useful; in the details of its spectrum, a variety of fundamentally different physical phenomena can be studied, from spin interactions in its fine structure to nuclear structure in the Lamb shift (see [1] in this issue for a summary). As antihydrogen is a pure antimatter atomic system, it is a CPT conjugate of hydrogen. Consequently, it can be used for direct CPT (charge, parity and time reversal) symmetry tests across all manner of atomic interactions present in its matter equivalent. ALPHA has made substantial progress on measuring properties with trapped antihydrogen through routine trapping of this exotic atom and the first measurements of its atomic transitions [2–4].

The question of antimatter and gravitation arose in the early part of the twentieth century with the idea of antigravity—namely that matter and antimatter would repel each other gravitationally. As a concept, this has largely been ruled out, but antimatter gravitation is held as an important system for use in testing the Einstein equivalence principle and non-Newtonian models of gravity [5]. Antihydrogen is a useful experimental system for performing equivalence tests owing to its complete composition of antimatter as well as its nominal charge neutrality, which reduces a large number of practical and fundamental sources of systematic error in measurements. As for what scale differences between matter and antimatter gravitation might manifest, modern theory runs the gambit between large violations being possible (approximately tens of per cent) to limits below 10^−5^% [6,7]. As this particular question has not been addressed empirically to date, it is paramount to measure the actual behaviour of antihydrogen to help guide and set limits on these various theoretical frameworks. In addition to the effort proposed by ALPHA and described below, this is considered a sufficiently important investigation that at least two other collaborations (AEgIS and GBAR) operating at the Antiproton Decelerator (AD) facility are actively pursuing antimatter gravitation measurements [8,9].

## 2. Antihydrogen trapping

Production of antihydrogen from cold positrons and antiprotons has been achieved by several groups over the years since 2001 [10–12]. A little under a decade later, ALPHA published the first trapping result, with a report by the ATRAP experiment the following year, ushering in the era of direct measurements of low-energy antihydrogen atoms [2,13].

**Figure 1.**
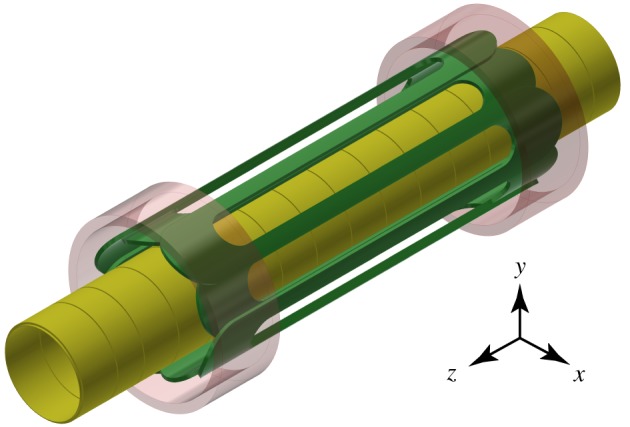
Example of an ALPHA hybrid non-neutral plasma/atom trap. The Penning–Malmberg trap is formed by applying potentials on cylindrical electrodes (yellow) and imposing a uniform background magnetic field ***B*** aligned with the cylindrical axis of symmetry (*z*-axis in this image). The magnetic trap is created with additional fields from two short superconducting mirror coils (red/pink) that are used to form two minima in the *z*-direction and a superconducting octupole (green) wrapped around the trapping volume to create a minimum in the transverse direction. With a nominal background field of 1 T, the two mirrors and octupoles are energized to make a trap depth of Δ|***B***|≈1 T. (Online version in colour.)

ALPHA’s approach to production of antihydrogen across all its experiments is to form it by direct combinations of antiproton and positron plasmas. Populations of cold positrons and antiprotons are co-confined in a Penning–Malmberg trap where they are manipulated in a variety of ways (figure 1). Approximately 30 million antiprotons are ejected from CERN’s Antiproton Decelerator at an energy of 5.3 MeV every hundred seconds and subsequently caught and cooled within a Penning trap. Recombination is achieved by directly overlapping the positron and antiproton populations. A superimposed Ioffe-like magnetic-minimum trap confines low-field-seeking antihydrogen atoms if they have a total kinetic energy *E* lower than the effective trapping potential depth 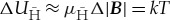 (*T*∼540 mK for ground-state antihydrogen in ALPHA). Detection of antihydrogen is achieved through the use of a silicon vertex detector that is able to locate its antiproton annihilations to within approximately 7 mm (see [14] for a detailed description of the original ALPHA trap system).

In the initial ALPHA trapping result, antihydrogen was detected by first clearing any remaining charged particles from the trap by applying a uniform clearing electric field across the trap (a linear electrostatic potential *ϕ*), and then identifying the antiproton annihilations appearing within the trap volume during the approximately 30 ms shutdown time of the superconducting magnet system as being indicative of the release of trapped antihydrogen [2].

Before ALPHA published the first trapping result in 2010, careful consideration was given to possible signal contamination by annihilations from antiprotons which remained mirror-trapped by the atom trap magnetic fields [15]. In the full trap, including the clearing potential, an adiabatic antiproton with initial kinetic energy *E*_⊥_0__ perpendicular to ***B***_0_ will experience a potential on-axis of
2.1
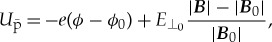
where *ϕ*_0_ and ***B***_0_ are the electrostatic potential and magnetic field that the particle experiences at the centre of the trapping potential. Even with the maximum clearing potential applied, a category of antiprotons will remain trapped by the mirror potential if it has sufficiently high *E*_⊥_0__. These mirror-trapped antiprotons would be released over the course of the magnet ramp-down, contaminating the antihydrogen signal.

As the dynamics of the charged antiprotons are expected to be more complex than simple adiabatic scaling considerations could predict, a code was developed to solve the full equations of motion for antiprotons and antihydrogen atoms in the time-varying trap fields, and experiments were conducted using antiprotons with intentionally large *E*_⊥_0__ [2,15]. In the experimental protocols used, the clearing electric field was left on during magnet ramp-down to distinguish populations of trapped antiprotons (which would tend to systematically deflect in accordance with the direction of the electric field) from antihydrogen atoms (which would tend to annihilate symmetrically in the trap). The inconsistency between the putative trapped antihydrogen atoms from the expected annihilation pattern of the antiprotons allowed high certainty on the detection of the released anti-atoms. In fact, this technique is used by ALPHA to date to qualify all our trapping protocols against this possible background.

## 3. Measuring potentials with antihydrogen orbits

In a broader context, this investigation, in distinguishing mirror-trapped antiprotons from antihydrogen atoms, launched a pursuit of what other physics questions could be addressed through careful consideration of atom orbits within an ALPHA-like atom trap. Here, we consider two avenues that can be tackled in this manner: (a) whether antihydrogen atoms have charge and (b) whether antihydrogen’s acceleration in a magnetic field differs from that of hydrogen.

### (a) Antihydrogen charge measurements

**Figure 2.**
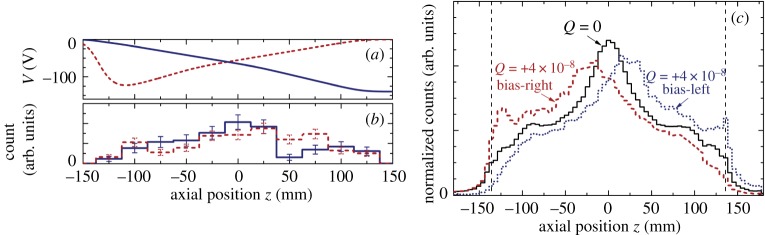
(*a*) Plot of the electrostatic potential for generating fields *E*_*L*_ (blue) and *E*_*R*_ (dashed red). (*b*) Antihydrogen annihilation distributions for left (blue) and right (dashed red) biases. (*c*) Simulated annihilation distributions for *Q*=0 (black curve) and (*Q*=+4×10^−8^) under the applied left (dotted blue) and right (dashed red) bias curves. Plots adapted from [16].

The first question in some ways came about as a natural extension of the kind of experiment we were already conducting. As we already performed all our antihydrogen detection in the presence of a bias electric field *E*_bias_, we were in a position to test the hypothesis that antihydrogen itself had a charge anomaly *Qe*, where *e* is the fundamental charge unit and *Q* is a constant. During the magnet ramp-down, the deflection of hypothetically charged antihydrogen atoms can be both analysed from simple considerations and calculated in detail with the extensive orbit simulations developed previously. We consider a modified potential for antihydrogen atoms in the trap of the form
3.1
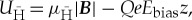
where *z* is the longitudinal coordinate in the trap and *E*_bias_ is a constant electric field pointing along the trap axis. The half-difference between the average *z*-position of the antihydrogen annihilations, 〈*z*〉_Δ_, is calculated from the two distributions generated with a bias electric field *E*_*R*_ pointing in the positive direction and *E*_*L*_ pointing in the negative. Under this protocol, a charge anomaly of antihydrogen can be calculated measured from
3.2
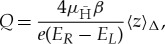
where *β* is a geometric factor based on the quadratic expansion of the magnetic field in the trap. Figure 2*a*,*b* shows plots of the bias potentials used to generate *E*_*R*_ and *E*_*L*_ and the corresponding annihilation distributions measured.

This simple calculation was compared against detailed simulations of the orbits that variously charged antihydrogen atoms take while escaping during the antihydrogen detection ramp and found to be in surprisingly good agreement with the simple model. Figure 2*c* shows examples of simulated distributions for various values of *Q*. Using an extensive survey of *Q*, a map from experiment 〈*z*〉_Δ_ to *Q* could be calculated. An *ex post facto* analysis of trapping data from the ALPHA-1 trapping series yielded a 〈*z*〉_Δ_ of 4.1±3.4 mm, setting a limit on *Q* of (−1.3±1.1±0.4)×10^−8^, including statistical and systematic errors [16].

This method of charge measurement from deflection is at the limit for what could be accomplished with this measurement in ALPHA-1 and -2. However, the work inspired an intentional measurement of antihydrogen charge neutrality [17]. In this experiment, we deliberately applied a series of stochastic electrostatic kicks intended to heat (charged) antihydrogen atoms out of the trap. Using this technique, we compared the measured survival rate against survival rates of simulated charged antihydrogen atoms subject to the same series of kicks. In this way, we avoided practical limits on bias field strength and uncertainties in detector and trap geometry and were able to improve the limit on |*Q*| to less than 0.71×10^−9^. Further application of this experimental protocol could improve this measurement by several orders of magnitude [17].

The charge neutrality investigation exemplifies one way in which properties of this atomic system can be investigated through careful modelling, control and measurement of its orbits in our trapping system.

### (b) Antihydrogen gravitation proof-of-principle measurement

The second such question we addressed along these lines was that of antimatter gravitation [18]. In this case, we considered the dynamics of antihydrogen in our trap under the action of a modified gravitational force for antihydrogen: 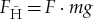, where *F* is interpreted as the ratio of antihydrogen’s gravitational to inertial mass. We consider a new potential for antihydrogen atoms in our trap:
3.3
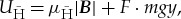
where *y* is the vertical coordinate in our atom trap.

For the fields typical of the ALPHA-1 and -2 magnets, the antihydrogen magnetic moment 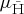 is approximately 0.670 K T^−1^, and results in a trap depth of approximately 540 mK for antihydrogen atoms born at the centre of the magnetic volume. In these traps, antihydrogen atoms are confined vertically primarily by the field gradient of the octupole, making the gravitational potential difference across the trap approximately 50 μK over the approximately 44 mm height of the trap. Antihydrogen will tend to be deflected vertically by the additional gravitational dependence, which in principle can be measured with our position-sensitive detector.

**Figure 3.**
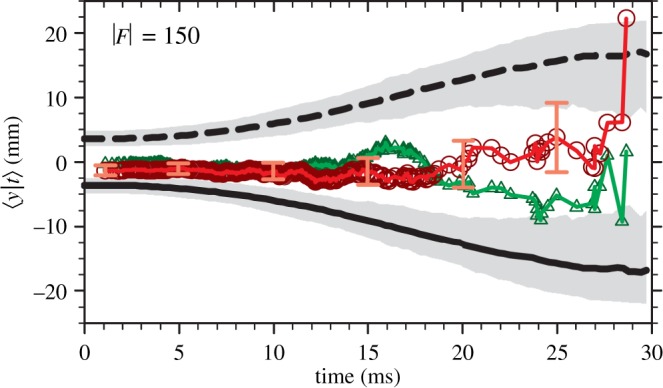
Reverse cumulative average of antihydrogen annihilation positions 〈*x*|*t*〉 (green triangles) and 〈*y*|*t*〉 (red circles) for 434 antihydrogen events from the ALPHA-1 experiment. Simulations of the RCA for *F*=+150 (dashed black line) and *F*=−150 (solid black). Grey bands show the 90% confidence intervals from the simulations. Plot adapted from [18]. (Online version in colour.)

The gravitational analysis involved calculating the reverse cumulative averages (RCA) 〈*x*|*t*〉 and 〈*y*|*t*〉 for antihydrogen annihilation positions as a function of time *t* during the trap ramp-down. This choice of measure aimed to weigh the fact that events occurring at later times during the magnet ramp, though fewer in number, will deflect more by the gravitational potential as they possess a lower energy. A detailed comparison between the experimental distribution measured in ALPHA-1 against extensive simulations for different test values of *F* was made. Figure 3 shows an example of the RCA of annihilation positions compared against simulations for *F*=±150. The RCA distributions from the real data are tested for incompatibility with simulated data in order to create a bound on *F*. With this approach, we excluded *F*<−12 and *F*>75 at the 95% certainty level. Worst-case scenarios for possible systematic errors moved these bounds to *F*<−65 and *F*>110 [18].

As a proof-of-principle experiment, this analysis activity served to identify the key areas of improvement required to achieve a better measurement on *F*. The dataset used in the above analysis included 434 antihydrogen atoms that are consistent with sampling from the cold (less than 540 mK tail of an approx. 50 K distribution). Obviously, a larger sample set would improve statistical significance, as would a lower-temperature antihydrogen population (several limits on this are described in [18]); however, these would soon be swamped by systematic effects that ALPHA-1 and -2 were not designed to minimize.

## 4. ALPHA-g: an apparatus for measuring gravitation

**Figure 4.**
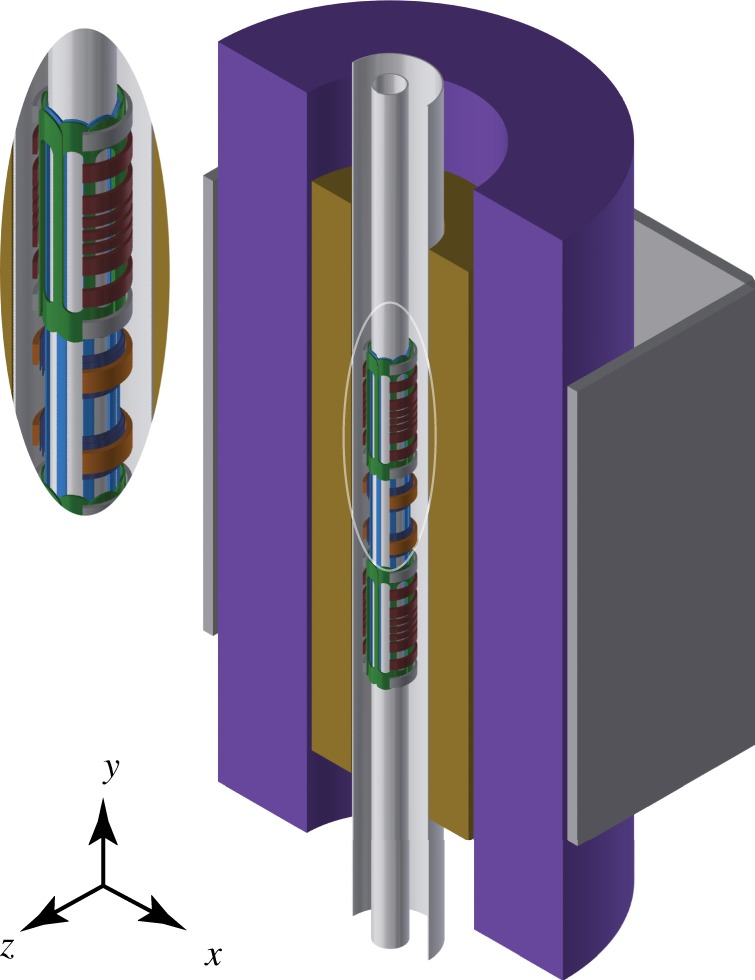
Schematic of the ALPHA-g magnet system, with its cylindrical axis of symmetry oriented in the vertical (*y*) direction. An external solenoid (purple) generates the uniform solenoidal field required for internal Penning traps and operation of the radial time projection chamber detector (gold). Inset shows details on the upper and precision trap. Two independent atom traps surrounding Penning traps are generated by a set of seven mirror coils (red) and a short octupole (green). A precise analysis trap is formed between the two dark orange coils and a long octupole (blue). Adiabatic transport of antihydrogen atoms between trapping can be accomplished through sequencing four transfer mirror coils (grey). External magnetic error fields can be corrected through rectangular correction coil panels (dark grey). Possible trapping regions range in length from approximately 280 mm (single end atom trap) up to 1.3 m (between the extrema of the two end traps). (Online version in colour.)

With the experience gained through these analyses and measurements, we designed a new atom trap for use in measuring antimatter gravitation. ALPHA-g consists of a vertically oriented apparatus consisting of two symmetric atom and Penning trap arrangements with a high-precision region in the centre. Surrounding the cryostat is a radial time projection chamber tracking detector used for locating antiproton annihilations within the trapping volume. Figure 4 shows the layout of magnets in the planned ALPHA-g apparatus. The symmetry of the design is aimed towards conducting equivalent experiments on either end of the trap to set limits on or cancel systematic construction and detection errors.

In the ALPHA-1 and ALPHA-2 traps, magnetic potential in the *y*-direction is essentially fixed by the construction of the octupole magnet, with no provision for compensating systematic errors either in construction or from external fields. With a vertically oriented trap, the magnetic potential in the vertical direction can be tuned via independent control of the trap mirror currents. It is expected that, for a first measurement, the magnetic fields from the various coils can be directly measured on-axis using techniques similar to those outlined in [19].

With this arrangement, it makes a rough measurement of the sign of *F* straightforward. For sub-540 mK antihydrogen in a trap roughly 280 mm tall, atoms will bounce over the height approximately 1000 times in 10 s. If we shut the trap down during that time, the field will change by Δ*B*≈(1 T)×(5 ms/10 s per bounce)=5 G per bounce. The magnetic potential change for the atom during this orbit is approximately 670 mK T^−1^×5 G=340 μK. This energy corresponds to a gravitational potential difference for hydrogen over a distance of approximately 280 mm. As this is more or less the geometry of the trap, it means that, as an atom is bouncing back and forth, it will tend to leak out of the bottom of the trap instead of the top during a ramp (for *F*=1).

This is an ideal picture, as the real field profiles result in atomic orbits that are chaotic and hence do not lend themselves to this sort of immediate scrutiny. However, simulations verify that this scaling argument holds in practice, and that, with this described experiment, antihydrogen atoms will exit towards the bottom approximately 71% of the time. This makes it rather efficient to conduct hypothesis testing on the sign of |*F*|≈1, with a few hundred antihydrogen annihilation events—a data rate which is presently achievable during a single 8 h shift on ALPHA-2 [20].

Obviously this is an overly simplistic picture, and a measurement on the value of *F* will require high-precision characterization and manipulation of the magnetic field, as well as a colder population of antihydrogen; the long-term programme for ALPHA-g is to develop these techniques. We expect that we will be able to perform *in situ* magnetometry using microwave spectroscopy and electron-cyclotron resonance measurements at the sub-50 μT level on the axis of the experiment using techniques inspired by Ahmadi *et al*. [3] and Amole *et al*. [19]. However, we are also developing nuclear magnetic resonance-based magnetometry probes to generate high-resolution field maps of our magnet system which may ultimately be required to understand systematic errors in measurement of *F* at the 1% level.

In addition to improved magnetometry, we will need to reduce the temperature of our antihydrogen distribution. There are two methods which are provisioned for ALPHA-g. The first is adiabatically cooling the antihydrogen distribution. The arrangement of magnets in figure 4 is such that antihydrogen can be formed in a longitudinally short trap and expanded out to the longer analysis region. Depending on the experimental conditions, one might expect lowering of the temperature by a factor of two or so. The development of a Lyman-*α* laser system to excite the 1S to 2P transition in antihydrogen and demonstration of an experimental protocol in ALPHA-2 are presently under way. A long-term deployment of a laser cooling scheme similar to that proposed in [21] is part of the ALPHA-g project. With a combination of adiabatic and laser cooling, we hope to achieve a sub-50 mK population of antihydrogen. We estimate that the first version of ALPHA-g will be capable of measuring *F* at the 1% level with all these developments in place.

## 5. Conclusion and outlook

ALPHA has developed a set of computational tools for dynamical tracking of antihydrogen and charged particle orbits in the relatively large traps used in its antihydrogen experiments. ALPHA-g represents a culmination of this effort in that it will fundamentally use the orbits of atoms in the trap and their consequent escape trajectories to measure the effective force of gravity on antihydrogen. Ultimately, the precision limit on this experiment will be in the systematic characterization of the magnetic fields that the atoms experience. Additionally, details of the atom orbits may also contribute to systematics. Consequently, a significant portion of the ALPHA-g experimental work will focus on developing experimental protocols to verify orbit calculations to both understand and control the antihydrogen distributions in the final high-precision gravity measurements.

Ultimately, uncertainties in the control and global knowledge of the magnetic field will limit the measurements performed by this first incarnation of ALPHA-g. With an improved trapping rate and production of ever-colder antihydrogen, it may be possible to conduct gravitational measurements using atomic interferometry techniques. Light-pulse matter-wave interferometry stands as one of the most precise tools for performing absolute measurements of the local gravitational potential, with precision on the parts-per-billion level in matter (see [22] for a review). Bringing an experimental tool such as this to bear on antihydrogen would be a substantial step forwards in possible antimatter gravitation studies. With the intended developments from the ALPHA-g programme, one could envisage precision at the parts-per-million level using a scheme like that in [23] for antihydrogen in an ALPHA-like trap. Inclusion of such a scheme would require significant reorganization of the internal superconducting magnet system, though the mechanical design of the cryostat is modular enough to make such a rearrangement conceivable in a future upgrade to ALPHA-g. Higher levels of precision for both this and other measurements in ALPHA will require a migration to magnetic field-free techniques.

It should be noted that with the increased antihydrogen trapping rate enjoyed presently at ALPHA, and prospects for future improvements from techniques like [24], it appears plausible to conduct instances of all these measurements over the course of one day or even fractions of a day. This means that there is significant hope that gravitational measurements could be performed systematically at various times of the day and throughout the year to test velocity and frame-orientation dependence on the gravitational acceleration. This approach to data collection can set limits on a larger set of terms which are included in theories such as the standard model extension that allow for CPT and Lorentz violation (see [25] for a review).

Finally, the anticipated upgrade of the AD facility to include ELENA has been a great boon to the antihydrogen community in general and this project specifically. For ALPHA, it is improved access throughout the beam period which is likely to be of most immediate benefit to us. We intend to operate ALPHA-g (to focus on gravitational acceleration) and ALPHA-2 (spectroscopic measurements) in tandem. The increased 24 h beam availability to experiments is critical in enabling this mode of operation, and indeed was one of the driving factors in the collaboration’s decision to initiate the ALPHA-g project when we did. Further, with uniform availability of antiproton time, it will be possible to schedule measurements across the day and across the time of year as described above. Having said that, with an anticipated 50-fold increase in antiproton number per spill, it will be simpler for us to develop experimental techniques which can effectively use this vastly increased resource and usher in a step change in trapping rate. We are eager to carry out the physics goals of ALPHA-g, ALPHA-2 and beyond in the epoch of ELENA.

## Data accessibility

This article has no additional data.
